# Change in the SBCCV’s Medical Residency Program - Perspectives of the Specialty and Challenges of Young Surgeons

**DOI:** 10.21470/1678-9741-2022-0957

**Published:** 2022

**Authors:** Gilberto Venossi Barbosa

**Affiliations:** 1 Faculdade de Medicina da Universidade Federal do Rio Grande do Sul, Porto Alegre, Rio Grande do Sul, Brazil

The invitation for analysis and discussion of these relevant issues was made to me by the Associação Brasileira de Residentes em Cirurgia Cardiovascular (ABRECCV). With 39 years of experience as a preceptor and 27 years as coordinator of the commission to prepare the teaching and training programs of the Sociedade Brasileira de Cirurgia Cardiovascular (SBCCV), I accepted this honorable mission.

The SBCCV’s medical residency program (MRP) is a living organism that has a past, present, and future, and is modified by the incorporation of advances in the specialty. The first step in changing a training program is to identify why this was necessary. This task is essential, so we don’t make mistakes, and it necessarily leads us to know the past, and whoever doesn’t will be giving up having the present and the future^[1]^. In the past, we sailed in calm waters, operating on patients with various structural heart diseases with surgical indication established by medical science and contained in current guidelines, when new facts appeared with great potential for changing directions. Cardiologists, stimulated by technological advances in their field, have moved from diagnosis to interventions, taking on a new role as interventional cardiologists. With the mastery of these skills, they invested in the treatment of ischemic heart disease, treating some lesions of the coronary arteries using angioplasty and intraluminal stents. They started to defend these methods with the concept of being less invasive compared to the greater exposure of conventional surgery. Several structured studies comparing the two approaches provoked different interpretations by the groups involved. At the end of the studies, the analysis of primary outcomes maintained the surgery with the best prognosis, even treating patients with higher risk scores and multi-vessel and more complex lesions, and the systematic use of anastomosis of the left internal thoracic artery in the anterior interventricular artery. Even though these results are included in all guidelines of the main specialty societies, interventional cardiology continues to treat a significant portion of these patients^[2]^. These facts, continued, provoked a huge reduction in surgical procedures of myocardial revascularization, the most frequent surgical procedure in our specialty^[3]^. The SBCCV’s MRP in Cardiovascular Surgery was developed in accordance with Resolution 1785/2006 of the Conselho Federal de Medicina (CFM) of the Associação Médica Brasileira (AMB) and the Comissão Nacional de Residência Médica (CNRM) of the Ministério da Educação (MEC). In that same year, this commission, through Resolution number 2 (of 05/17/2006), admitted it as a National Medical Residency Program in Cardiovascular Surgery, but with two years of prerequisite in general surgery, making the training period extremely long. The immediate reflection of these decisions was the alarming drop in candidates for training in the specialty, occupying only 24.2% of the 323 vacancies offered each year, and it became urgent to reverse this disastrous situation. We had a previous successful experience, which continues to this day, with SBCCV’s Teaching and Training Centers, which have always been of direct access and with a significant number of candidates, proving the negative impact of the inclusion of the prerequisite^[4^,^5]^. Then, a wave of innovation using new technologies introduced a series of procedures with less invasive potential, via mini-access and percutaneous catheters, occupying an important space in the treatment of valvular, aortic, and congenital heart diseases, implants of mechanical circulatory assistance devices, and in the treatment of arrhythmias. In other words, they reached the full spectrum of our performance. It has become urgent to acquire expertise in these new forms of treatment^[6]^. Experienced surgeons with a broad vision of the future encouraged their peers to acquire skills in catheter procedures^[7^,^8]^. With the support of large international institutions, including Brazil, they developed a set of new minimally invasive techniques increasing the therapeutic arsenal of surgery benefiting a significant number of patients^[9^-^11]^. The reformulation of the CNRM collegiate, assuming the surgeon Rosane Leite de Melo as executive secretary, accepted the SBCCV’s arguments, approving the direct access program excluding the prerequisite of general surgery and establishing five years for completion of the residency in the specialty^[12]^. The president of SBCCV from 2016 to 2017, professor Fábio Jatene, with dedication and pertinacity led this important and decisive mission, assisted by Professors Henrique Murad, Renato Kalil, and Rui Almeida, members of its board, and Doctor Rosane Leite de Melo, executive secretary of MEC’s CNRM, who played an effective role in this outcome. It was a big and decisive step, because, immediately, the interest in attending our residency was renewed, translated by the expressive increase of candidates. Brazilian cardiovascular surgery is recognized by the international community for the skill and creativity of its surgeons, and for the contributions of new surgical techniques routinely used by surgeons around the world. This far-reaching legacy initiated by our pioneers is kept alive by the dedicated work of new and creative actors^[5]^.

## Analysis of the New Program Changes

The pedagogical structure of this new teaching and training program assimilated these transformative innovations with the aim of producing a new model of cardiovascular surgeon. It comprehensively includes all theoretical and practical cognitive framework from diagnosis to surgical treatment by mini-invasive and conventional techniques. With a clear description of the primary and secondary objectives and a careful selection of skills, it has become a powerful instrument for permanent consultation, guiding the performance desired by the resident^[13]^. The new format opened up a generous space for mastering the essential procedures to compete proficiently in the competitive job market. The strong emphasis on programmed internships in Cardiology, Hemodynamics, Thoracic Surgery, and Vascular Surgery, repeated in the initial four years, plays a powerful role in acquiring new skills and competences. So, we have an excellent program whose integrality of execution will only be possible in hospital services that have, in addition to basic cardiovascular surgery, the full spectrum of subspecialties. This profile includes surgery for congenital heart diseases, heart transplantation, implantation of circulatory assist devices, valve and coronary surgery using video-assisted or robotic mini-access, and endovascular treatment of diseases of the aorta and its branches using percutaneous endoprostheses^[14]^. For this to be possible, the institution must have qualified professionals, state-of-the-art imaging equipment, hybrid rooms, multidisciplinary teams, and preceptors with full mastery of these technologies, with a satisfactory volume of cases^[15]^. We have few institutions with this profile in Brazil. The new program, sensitive to these facts, offers alternatives to solve this scenario through an academic mobility process, which allows and encourages the resident to seek new skills and competences where they are available, during their training period or in additional years of training. In Brazil, training in basic cardiovascular surgery, without considering all the advances of new procedures, is performed with a good level of proficiency in all services with accredited residency programs. Residents from these services have a reasonable job market, as all surgeons, including here those who will seek new skills, and they need this essential basic training. After all, not every patient is a candidate for mini-invasive techniques due to various anatomical factors, among other impediments. The distribution of the workload covering various activities is an important factor that facilitates the performance of residents. This program complies with the regulations issued by the CNRM, which requires 2888 hours per year, including 10% to 20% for theoretical activities and 80% to 90% for in-service training. The SBCCV’s commission, in an innovative process, redistributed these 14,440 hours over five years, placing emphasis on tutored teaching in the operating room, allocating 60% (8640 hours), and 5% (720 hours) in the operative technique laboratory for training in simulators. Although all the activities of the program are very useful and necessary, none surpasses in importance the operative act in the operating room with the supervision of the mentor^[16]^.

The sum of hours spent by residents in this activity is one of the most expressive markers of the program’s quality. Mastery of theoretical cognitive content enables residents to know how to say and mastery of practical content enables them to know how to do. The curriculum of this new program indicates all these steps in order to prepare cardiovascular surgeons in these areas. The competence matrix set out for each year of residency, defining the general and specific objectives, constitutes a powerful instrument of the expected performance at each stage. Observing carefully, they will identify that each year from the first to the fifth has a pedagogical logic of progressivity with greater exposure both in the offer of theoretical content and in the levels of practical activities^[13]^. The first year has been completely restructured, the resident is exposed to the fundamentals of cardiovascular surgery through rotations with an emphasis on diagnostic methods in clinical cardiology, hemodynamics, imaging methods, vascular surgery, thoracic surgery, general surgery, extracorporeal circulation, and intensive care unit. The objective of studying these areas is to provide a basic and general view of the universe of the diagnostic and therapeutic arsenal available for the management of patients. From the second to the fourth year, the primary objective is to offer, in a sequential and progressive way, the deepening of the cognitive domain of cardiovascular diseases and the forms of conventional and mini-invasive surgical treatment. I identify that there is a high concentration of theoretical content, which to be assimilated will require extra hours of study invading the moments destined for rest and leisure. The fourth and fifth years were planned by placing a strong emphasis on the field of practical work, becoming the engine that enshrines know-how. The offer in the second semester of these years for the resident to study full-time in an area of interest, aiming to consolidate or acquire new skills, constitutes an effective tool to seek proficiency, signaling one of the great novelties of this program. This performance will only be possible with the effective implementation of a robust mobility system, through scheduled rotations between the services enabled by the SBCCV or the CNRM. At the end of five years of training, some areas of complex procedures will require an additional year or two of training to acquire proficiency in their complete domain. In our view, they can be carried out through fellowship courses in Brazil or at international centers certified through agreements signed with the SBCCV^[17]^. The opinion of residents who complete the five regimental years and of the preceptors of this new program will be a valuable tool to correct the trajectory, considering what is feasible and where are the difficulties and failures of its pedagogical structure. The individual performance evaluation system is well structured; however, I think it could take place every six months, simplifying the process. It would maintain the stated virtues, allowing the resident to correct directions when necessary. The level of proficiency of the graduates of the program, working in the community, will give us the answer we want to know, if we were able to produce a new model of cardiovascular surgeon.

## The Perspectives of the Specialty

The Brazilian population shows a substantial increase in the age group of people over 65 years of age, where ischemic, valvular, and vascular degenerative cardiovascular diseases are more prevalent, more complex, and at greater risk for invasive interventions. These patients are special candidates for the use of less invasive procedures performed by multidisciplinary teams through mini-access or percutaneous catheters guided by imaging equipment in hybrid rooms with a significant reduction in operative risk. This scenario is no longer future, it is happening now and with solid signs of growth and application also in cases of medium risk in younger patients (below 60 years of age) with promising results. New technologies in full development are signaling the need for surgeons with a new training profile^[17^,^18]^. In recent data from the SBCCV, it was found that 55% of Brazilian cardiovascular surgeons in activity are over 60 years of age. With rare exceptions, most of them will continue to competently perform conventional surgery that covers the entire spectrum of cardiovascular diseases. Younger surgeons who are proficient in the new procedures will have ample workspace. Other options are to invest in subspecialties where there is a chronic shortage of surgeons and high demand from patients, such as in the treatment of congenital heart diseases where the waiting list is immense. In Brazil, around 80% of the 27 thousand children with heart disease that are born each year should be treated, but only half are operated on, the rest increase the waiting list with high mortality. In our country, there are 68 services that treat these patients, and it is imperative to double the number of surgeons in this important subspecialty. Another sensitive areas with a shortage of services and surgeons is the treatment of advanced heart failure through heart transplantation and implantation of circulatory support devices and the treatment of arrhythmias through the implantation of multi-site pacemakers and cardioverter/defibrillators. So, there is a wide field of work for surgeons with mastery in the treatment of these patients. But only those qualified to know how to do it will compete for this important work space^[14]^.

## The Challenges of New Surgeons

The first challenge is to accept the paradigms that have transformed the specialty by embracing and understanding the changes. It is not easy to radically change from thoracotomy and suture techniques to mini-access or percutaneous procedures with guides, catheters, and images^[13]^. An important law of life determines that survival is not the preserve of the strongest, but of those who have the greatest capacity to adapt. It will be essential to become competent in the execution of these new procedures based on new technologies^[18^,^19]^. This will require more training time of one to two years invested in the subspecialties of cardiovascular surgery after completion of residency^[15]^. The universe of this scenario no longer allows one to be a specialist in several areas of the specialty, the volume of new information and new techniques changes every three months and requires in-depth, very high competence, and absolute mastery of the procedures of the chosen sub area^[19]^. It is necessary to be part of a Heart Team not as an expendable sidekick but as an essential member in decision making. In the management of high-risk patients by a multidisciplinary team, performed in hybrid rooms, it is necessary to be one of the main actors respected for their competence and total mastery of the surgical act and not seen as a secondary professional in the process^[13]^. New surgeons should maintain the habit of permanent study and use the continuing medical education courses provided by medical societies, industry, and institutions as an updating tool^[20]^. Also, include systematic training in very high-fidelity simulators to maintain a high level of proficiency, especially when technical variants in their area of expertise arise^[21]^. Finally, do not forget that by training they are the only professional in this scenario qualified to treat complications that require conversion to the open method^[22]^. Those qualified for these procedures will have a broad and heated job market, due to assimilation into traditional service teams, due to a significant increase in demand, and due to the choice and preference of patients.

I conclude this text by thanking all preceptors and mentors that for many years with total dedication, competence, and sense of responsibility continue to train Brazilian surgeons.
